# Knowledge of Stakeholders in the Livestock Industries of East and Southeast Asia about Welfare during Transport and Slaughter and Its Relation to Their Attitudes to Improving Animal Welfare

**DOI:** 10.3390/ani9030099

**Published:** 2019-03-19

**Authors:** Ihab Erian, Michelle Sinclair, Clive J. C. Phillips

**Affiliations:** Centre for Animal Welfare and Ethics, School of Veterinary Science, University of Queensland, Gatton, QLD 4343, Australia; ihab.erian@gmail.com (I.E.); m.sinclair6@uq.edu.au (M.S.)

**Keywords:** animal welfare, Asia, knowledge, slaughter, transport, training

## Abstract

**Simple Summary:**

The potential to improve stakeholders’ knowledge of animal welfare in the livestock industries through training programs and its influence on their attitudes to livestock welfare is unclear. Stakeholders in East and Southeast Asia responded to a questionnaire on their knowledge of animal welfare considerations during livestock transport and slaughter, as well as indicating their attitudes towards the welfare of livestock at these times. They then received training, after which their knowledge scores increased. Knowledge scores had few connections to attitudes, but whether the respondents were certain or not about their attitudes to livestock welfare was most likely to have the strongest correlation to knowledge. Regional differences were evident and suggested that these differences should be considered in future training provisions.

**Abstract:**

The World Organisation for Animal Health (OIE) sets standards and guidelines for international animal welfare for the international livestock trade. The growing economic advancement in the East and Southeast Asian region suggested the potential benefit of a research study to examine stakeholders’ understanding of animal welfare during the transport and slaughter of livestock. A survey of stakeholders’ knowledge of livestock welfare in the transport and slaughter industries was conducted in four Southeast Asian countries, Malaysia, China, Vietnam and Thailand, in association with trainer and stakeholder workshops conducted in each country. The attitudes of participants towards animal welfare during slaughter and transport were also identified. Knowledge scores were in accordance with the respondents’ assessment of their own knowledge level. The biggest knowledge improvement was among Thai respondents, who tended to be younger and less experienced than in other countries. The respondents with the biggest improvement in knowledge scores were most likely to be involved in the dairy industry and least likely to be involved in the sheep and goat industries, with meat processors and those involved in pig or poultry production intermediate. The respondents who obtained their knowledge from multiple sources had most knowledge, but it increased the least after training. Connections between attitudes to improving animal welfare and knowledge were limited, being mainly confined to ambivalent responses about their attitudes. The study suggests that knowledge can be improved in animal welfare training programs focused on livestock welfare around transport and slaughter, but that local cultural backgrounds must be considered in designing the program.

## 1. Introduction

Animal welfare issues are recognized as an important concern associated with animal production in many countries, particularly those with existing animal welfare policies, legislation and public awareness [1]. It is important to also recognize that there are significant differences in attitudes to animal welfare issues between regions [2]. With the current trend for the expansion of animal production in developing countries, livestock legislation is beginning to be promoted internationally through animal welfare codes of practice and minimal animal welfare standards. In 2002, the World Organisation for Animal Health (OIE) began the process of creating animal welfare standards, which are largely derived from scientific and technical knowledge. Such knowledge has been developed through informal and formal processes [3], in particular the acquisition of facts, theories and ideas, through education, reading from reliable sources, peers, consultation and the media [4].

The theory of Planned Behavior acknowledges that knowledge and attitudes are important elements of changing behavior towards ethical animal welfare practices [5]. The extent of knowledge, the role and relevance of such knowledge and the complexity of the knowledge are all acknowledged to have an effect on attitudes and behavior [6]. The theory has been widely used, for example to predict alcohol consumption among school students [7], to change the behavior of employees on construction sites so that waste was reduced [8], to predict intentions to care for patients with alcohol dependence by nursing students [9] and to assess the importance of self-belief for developing ways to rectify alcohol problems [10]. It is acknowledged that knowledge alone is insufficient to change beliefs, and positive behavior strategies and regular checks are needed to increase compliance with the procedures (e.g., of hand hygiene in nursing students [11]). Other research has demonstrated the necessity of understanding the relationships between planned behavior and attitudes (e.g., in perceptions of workplace health and safety) [12].

The OIE Regional Animal Welfare Strategies (RAWS) aim to facilitate the regional implementation of the Terrestrial Animal Code, (Section 7 Animal Welfare), which presents animal welfare guidelines for safe international trade in terrestrial animals and their products, that has been adopted by the OIE member states. A regional strategy was developed for Asia, the Far East and Oceania in 2008 to provide a vision for this region in which the welfare of animals is respected, promoted and incrementally advanced, concurrent with the pursuit of progress and socioeconomic development [13,14]. The achievement of this goal in the region is expected to require education in the form of training stakeholders in key aspects of welfare identified by the OIE standards.

Asia accounts for 39% of global meat production, with China producing almost twice as much meat globally as the second highest producer, the United States of America [15]. In 2016, China produced 76.4 million metric tons of meat (beef, pork and chicken), the second highest year on record [16] and driven by the fact that meat consumption in this region is rapidly increasing. Annual animal slaughter increased from 10.2 to 13.5 billion animals between 1996 and 2016 [17]. Domestic beef consumption increased by 111% from 3.5 million tons in 1996 to 7.3 million tons in 2015 [18]. The improvement of animal welfare practices in developing countries can also lead to improved product quality and increased export trade opportunities. There is also an important social element, since approximately 70% of the world’s poorest economies are tied to livestock industries, including many Asian countries [19], which have the advantage of cheap labor and land [20].

The recent animal welfare guidelines of the OIE have advocated a number of ways of influencing regional and global approaches to animal welfare standards, but particularly suggest the implementation of the OIE guidelines in legislation and education [13]. Apart from seeking to identify knowledge in the livestock industry stakeholders, there have been few attempts to understand public knowledge of animal production systems [21] and to discover their sources of information. One qualitative study used the concept of Planned Behavior theory to identify the most noticeable consumer beliefs regarding dairy products in the food markets [22]. These studies can be used to guide implementation of the OIE standards, as knowledge gaps can be identified and rectified. The recommendation of the OIE is for each country to implement animal welfare standards or introduce a “Code of Practice” for animal welfare conditions. However, there remain instances where developing Asian countries have accepted lower animal welfare standards of practice than would be acceptable in developed countries, e.g., [23]. Stakeholders in the livestock industries now have roles and responsibilities to effect changes in the way society treats and views animals [24] and in providing the care and conditions to make the animals contented, not just avoiding pain and stress [25].

This paper focused on the importance of improving local husbandry knowledge of, and attitude towards, animal welfare during transport and slaughter in four diverse countries in southeast Asia (the Federation of Malaya, hereafter referred to as Malaysia, the People’s Republic of China, hereafter China, the Socialist Republic of Vietnam, hereafter Vietnam, and the Kingdom of Thailand, hereafter Thailand). Attitudes included towards the effect of local laws, the effect of personal and religious beliefs and the importance of any improvement to workplace, community and peers. It was hypothesized that knowledge of the standards would improve attitudes towards animal welfare. The study also examined the cross-cultural differences between these countries and the way that the knowledge had been acquired. Previous work with these stakeholders has examined their intention and ability to enhance animal welfare [26], as well as the differences between the different countries and stakeholders in their attitudes to livestock welfare during transport and slaughter [27,28].

## 2. Materials and Methods

Human ethical clearance was obtained from the University of Queensland Ethics Committee (Reference Number 2015000059). The study used a quantitative questionnaire that was administered at training sessions in Malaysia, China, Vietnam and Thailand (Supplementary Materials Section 1), which addressed stakeholders’ knowledge of livestock production systems, with a focus on livestock welfare during transport and slaughter and their attitude towards livestock welfare. These countries were selected due to their future export potential for international livestock products within the next few decades and the diverse cultures they represented.

Four two-day ‘train the trainer’ workshops were conducted under the auspices of an Animal Welfare Standards Project (http://www.animalwelfarestandards.net/), one in each country. These were led by four international livestock experts in animal slaughter and livestock transport. The participants comprised 118 trainers (30 from Malaysia, 46 from China, 20 from Vietnam and 22 from Thailand) (Table 1), who were given access to a resource package with presentations in the local language (http://www.animalwelfarestandards.net/). The trainers were chosen by country coordinators on the basis of involvement in the livestock industry, slaughter or transport.

The questionnaire was also answered by 896 stakeholders (94 in Malaysia, 338 in China, 196 in Vietnam and 268 in Thailand), who were trained by the trainers in a series of forty-four one-day regional workshops (Table 2). All stakeholders received travel allowances, free lunch and per diem expenses. The project coordinators in each country were responsible for selecting participants for the workshops based on their involvement in the livestock slaughter or transport industries in the different capacities identified in the questionnaire. The workshop invitees included delegates from local OIE veterinary services, local animal welfare focal point personnel, and personnel working directly with livestock in the transport and or slaughter industries. Farmers, team leaders who supervise people who work directly with the animals, business owners in the livestock industries, business managers in the industry, veterinarians who treat animals or work for the government as advisors and university researchers were also invited. After completion of the training on the OIE standards in each country, the same knowledge questionnaire was reissued and answered by the majority of the participants.

The questions (Supplementary Materials Questionnaire Section 1) were based on the OIE standards for international farm animal welfare in addition to the animal welfare material contained in the presentations to participants by the international experts in each field at the ‘train the trainer’ workshops. A pilot survey with 10 respondents from all the countries was conducted through industry stakeholders.

The initial questionnaires were developed in English in collaboration with researchers, academics, international experts in the animal welfare domain and literature. Adjustments were made to avoid leading questions or a possible bias. The final questionnaire was translated to Bahasa, Mandarin, Vietnamese and Thai and then back-translated to English to ensure that the translated version in the local language was consistent with the original questionnaire. The questionnaire was delivered as a hard copy to all the trainers, in their local language. Participants were given a unique code to facilitate identification before and after the workshops.

The first section of the questionnaire comprised 18 questions that focused on the trainers’ knowledge of the OIE animal welfare standards and common local livestock practices in each country, with specific reference to livestock welfare during transport and slaughter (Supplementary Materials).

The second section examined the participants’ attitude to animal welfare during transport and slaughter, how satisfactory they believed animal welfare to be, whether any improvements at their workplace could be initiated by new legislation or by international monetary gains for their local products [26,27,28]. The attitude questions were grouped into four sections, personal assessment, community assessment, ability to make improvements, all separately identified for slaughter and transport, thereby covering the central components of the theory of Planned Behavior. The response to each question was ranked on a five-point Likert scale, from strongly disagree to strongly agree. The first eight questions focused on general attitudes to animal welfare. The second set of questions investigated the key factors influencing the stakeholders’ assessments of animal welfare during slaughter and transport, which included: personal beliefs, relevant laws, the importance of animal welfare within the workplace, community and peers, the benefits of improving animal welfare within the community, workplace and industry, in general and in terms of monetary gain. The thirteen questions also investigated the factors influencing the stakeholders’ evaluation, capacity to improve and sources of improvement of animal welfare practices during slaughter and transport [26,27,28]. The final section contained demographic questions: the participants’ country, their age, sex, religion and residential region, their role within their industry, how long they have been involved in the industry and how their knowledge was gained (formal, employment or otherwise).

### Statistical Analysis

The questionnaire response data was initially tabled in a Microsoft Excel 2013 spreadsheet for each country and then transferred to Minitab Version 17 for analysis. A total of 330 stakeholders (Malaysia 7, China 294, Vietnam 16 and Thailand 13) were excluded from the analysis because their data was incomplete, mostly due to a failure to redo the knowledge test after the training. Only invited attendees’ data were included in the analysis. A husbandry knowledge score (K score) was determined for each respondent from the total number that were correct, out of eighteen knowledge questions. The change in numerical distribution of K scores was examined after the completion of the workshops to determine the usefulness and benefits of the training sessions.

A principal component analysis of the respondents’ answers was undertaken first but demonstrated little evidence of clustering. A stepwise general linear model regression analysis assessed the significance of the relationships between the respondents’ demographic data, as the independent variables, and the distribution of the Likert scale responses for the total K score, as the dependent variable, using a Logit link function. Least square means are quoted, and Alpha values for parameters to enter or leave the equation were set at 0.1. Plots of residuals were examined to ensure that the correlated factors between attitude and knowledge questions and demographics approximated a normal distribution and all probability values were considered significant at *p* ≤ 0.05. Post hoc comparisons of individual means was by Tukey’s test. 

## 3. Results

The mean response time to answer the questionnaire was 33 min. A total of 683 male respondents (67.4%), 310 female respondents (30.6%) and 21 respondents who did not indicate their gender (2.0%) completed the workshops and questionnaires (Table 3). The gender balance appeared different in Thailand, being almost equal, whereas in the other three countries the majority were male. The most common age group among all the countries was 26–45 (*n* = 634; 62.5%), only 45 respondents (4.4%) were in the category 56–65 years of age. Thai respondents were also more likely to be under 25 and to have less than one year of experience than in other countries. The most common source of gaining knowledge was from formal qualifications in the livestock industry, either through a relevant degree or training course (*n* = 464; 45.8%), with 14.0% gaining their knowledge from hands-on experience through employment in the relevant livestock industry. Vietnamese respondents were more likely to indicate that they gained their knowledge from all possible sources. Slightly more respondents, 575 (56.7%), indicated that they lived most of their lives in metropolitan or urban areas than those who indicated they lived in rural areas, 416 (41.0%). Chinese were more likely to be from an urban zone than those from other countries. Of 998 respondents (98.4%) who identified a religious affiliation, 411 respondents (40.5%) were Buddhist, 393 (38.8%) did not follow a religion, 77 (7.6%) were Muslim and 38 (3.7%) were Christian. In Malaysia and Thailand, most indicated that they were moderately or very religious, but in China and Vietnam most said that they were not religious. 

The respondents’ roles in the livestock transport and slaughter industry were varied: 304 (30.0%) worked directly with animals, 238 (23.5%) were supervisors, team leaders, business owners or managers within the industry and 290 (28.6%) were veterinarians working “hands-on” in the field or in a government advisory role. Thais were more likely to be farmers and the Vietnamese were more likely to be vets. Three hundred and thirteen respondents (30.9%) regarded themselves as being experts or having good knowledge of their relevant livestock industry, but 222 respondents (21.9%) regarded themselves as having little or no knowledge in the livestock production systems regarding animal welfare standards during transport or slaughter.

The most common involvement of the respondents was in abattoirs or in meat processing facilities (*n* = 291, 28.7%), followed by the respondents involved in the pig (*n* = 193, 19%) and poultry (*n* = 161, 15.9%) production industries. The most common length of involvement in the relative industry was 2–5 years (*n* = 327, 32.2%). One hundred and eighty four (18.1%) had had 5–9 years involvement in their respective industries, and 149 respondents (14.7%) had had over 15 years.

### Respondents’ Husbandry Knowledge

The respondents’ self-assessed level of knowledge of livestock production systems was most commonly reported as some, little or no knowledge (*n* = 670, 66.1%) with fewer than 6.0% (*n* = 61) of respondents regarding their knowledge as expert. Very few Chinese or Vietnamese claimed little knowledge, but a significant proportion of Malaysian and Thai respondents did. Most gained their knowledge from training courses or relevant degrees (*n* = 464, 45.8%), with a significant number gaining their knowledge from personal interest such as the internet, television programs, journals and newspaper articles (*n* = 92, 9.1%) (Table 3).

Overall, in the pre-workshop knowledge test, Thai respondents had a lower proportion correct, compared with Malaysian and Chinese respondents (Malaysia, 3.80 ^a^; China, 3.44 ^a^, Vietnam 3.23 ^ab^, Thailand 2.80 ^b^, out of maximum 15, Standard Error of the Difference between two means, SED 0.221, F-value, 4.56, *p* = 0.004). However, the improvement in the number correct by the end of the workshop was greater in Thai respondents than in Chinese or Vietnamese respondents (Malaysia, +5.80 ^ab^; China, +5.50 ^b^, Vietnam +4.46 ^c^, Thailand +6.24 ^a^, SED 0.291, F-value, 5.05, *p* = 0.002).

## 4. Influencing Factors

### 4.1. Attitude Effects on Knowledge Score

There were six attitude questions that significantly influenced the respondents’ K scores (Table 4). Mostly, the difference related to greater or lesser K scores for those who neither agreed nor disagreed, compared with the other responses. For the following four attitude questions, the K score was lower: I intend to make improvements to welfare of animals in my care; the laws on animal slaughter and transport influence my assessment of their animal welfare (AW) at this time; my knowledge about animal slaughter and transport limits my ability to improve AW during transport; and changes prescribed by my company encourage me to change practices. For the following two attitude questions, the score was greater: vehicle design makes improvement to AW during transport difficult; and changes prescribed by my supervisor encourage me to change practices.

### 4.2. Attitude Effects on Change in Knowledge Score Post-Training

There were nine attitude questions that significantly influenced the respondents’ change in K scores (Table 5). Mostly, the differences again related to a greater or lesser score improvement for those who neither agreed nor disagreed, compared with the other responses. For the following attitude questions, the improvement was greater: welfare of transported animals is satisfactory; importance to my peers of factors influencing welfare of animals; and encouraged to change if prescribed by government. For the following two attitude questions, the improvement was less: encouraged to change if prescribed by law; and monetary gain influences my personal assessment of welfare. When the respondents strongly disagreed with the statement “Importance of welfare to peers influences ability to make improvement during slaughter”, the improvement in their knowledge score was very low. Those strongly agreeing that their personal beliefs influence their ability to make improvement during transport also had reduced. 

### 4.3. Demographic Effects on Knowledge Score

Apart from their country, there were two demographic questions that significantly influenced K scores. The respondents who considered that they had good knowledge of livestock production systems actually had higher K scores compared with those who said that they had just some knowledge (expert, *n* = 54, 3.36 ^ab^, good, *n* = 195, 3.78 ^a^, some, *n* = 296, 3.18 ^b^, little, *n* = 105, 3.29 ^ab^, none, *n* = 21, 2.97 ^ab^, SED 0.221, F-value 2.65, *p* = 0.03). In relation to where they got their knowledge, those who indicated that they got it from all the possible sources had a higher K score than those who indicated any one particular source (formal qualifications, *n* = 326, 3.27 ^b^, farm employment *n* = 102, 3.25 ^b^, personal interest (internet, journals, newspapers, TV), *n* = 67, 2.95 ^b^, friends and acquaintances, *n*= 47, 3.21 ^ab^, and all of these *n* = 129, 3.91 ^a^; SED 0.221, F-value 3.00, *p* = 0.02). 

### 4.4. Demographic Effects on Change in Knowledge Score Post-Training

Apart from their country, there were three demographic questions that significantly influenced the respondents’ increase in K scores. Age had a significant effect on K Score improvement, with this being highest in those over 65 and lowest in those 56–65 (18–25, *n* = 101, 5.60 ^b^, 26–35, *n* = 232, 5.44 ^b^, 36–45, *n* = 197, 4.97 ^bc^, 46–55, *n* = 95, 5.54 ^b^, 56–65, *n* = 23, 4.10 ^c^, >65, *n* = 13, 7.35 ^a^, SED 0.221, F-value 3.00, *p* = 0.01). In relation to where they got their knowledge, those who indicated that they got it from all the possible sources had a lower K score increase than those who indicated that they got their knowledge from formal qualifications or friends (formal qualifications, *n* = 321, 5.59 ^ab^, farm employment *n* = 103, 5.09 ^bc^, personal interest (internet, journals, newspapers, television), *n* = 65, 5.53 ^abc^, friends and acquaintances, *n* = 47, 6.34 ^a^, and all of these *n* = 125, 4.94 ^c^; SED 0.300, F-value 2.48, *p* = 0.04). In relation to the type of livestock industry, the respondents involved with the dairy industry had greater improvement than meat processors or those involved in the pig industry, which along with those involved in cattle, abattoirs and meat processors, in turn had greater improvement than those in the sheep and goat industries (cattle, *n* = 98, 6.12 ^ab^, dairy, *n* = 35, 6.71 ^a^, abattoir, *n* = 221, 5.77 ^ab^, sheep/goat, *n* = 30, 3.69 ^c^, poultry *n* = 101, 5.58 ^b^, meat processors, *n* = 25, 5.28 ^ab^, pigs, *n* = 151, 5.36 ^b^, SED 0.300, F-value 3.89, *p* = 0.001). 

## 5. Discussion

The measurement of knowledge in this study appears to have been successful, since scores broadly agreed with stakeholders’ self-rated knowledge assessment. Also, those citing multiple knowledge sources had greater knowledge scores but the lowest increase after training, as expected. The questions were chosen to be at a mixture of knowledge levels and to be generic to the four countries.

### Demographics

The recruitment and selection criteria for the trainers had some potential bias when the local participants were selected for workshops. The trainers were nominated by the local authority in each country, who were under instruction to choose trainers with extensive involvement in livestock slaughter or transport. Although the respondents at the workshops were selected by the prospective authorities in their countries, some selection bias may have occurred by not inviting or including legislative personnel and animal welfare regulators in the workshops.

The respondents from the four countries identified that the main source of gained knowledge was through training courses and relevant formal qualifications (*n* = 464, 45.8%) from professional institutions such as universities, agricultural and veterinary colleges, livestock processing companies and livestock transport bodies (Table 3). Training could be tailored to the local needs by exploring the gaps in husbandry knowledge of the OIE animal welfare recommendations by implementing a mechanism, in the form of questionnaires, to identify these needs and to ensure the success of the training to deliver the desired outcome and give local people an understanding of the OIE animal welfare recommendations [29]. The study also identified that the least likely source of gaining knowledge was through television programs, internet, academic journals, newspaper articles, friends and acquaintances (162, 16%), which could be attributed to the limited access of some respondents to multimedia means of dissemination of information (Table 3). There were just 70 respondents (*n* = 1014, 6.9%) who gained their animal welfare knowledge through their friends and acquaintances, and 92 respondents (9.1%) gained it through personal interest via internet, journals, newspaper articles and television programs. However, those who did gain knowledge from friends had a greater increase in K score. This supports the contention that the peer effect in the East and Southeast Asian region is a strong influence on disseminating knowledge [30]. Previous research regarding the relationship between knowledge and animal welfare issues found that knowledge supplied by animal protection organizations was the most credible source of knowledge; however, this is likely to be because the respondents were from these organizations [31]. Further research on the effect of peers on the increase of knowledge in similar cultural backgrounds could be beneficial for the success of animal welfare programs and industry engagement. Research of animal welfare knowledge of advocates [31] has also identified that original scientific literature was highly regarded, which accords with the recognition in our study that gaining animal welfare knowledge through formal education is likely to lead to increased receptivity to new knowledge. 

## 6. Respondents’ Husbandry Knowledge

The data collected from the respondents identified that the oldest respondents (>65 years) improved their knowledge most, even though the sample size was small. Human morality is recognized as increasing with age in Kohlberg’s progression of moral reasoning [32]. Also the respondents who are involved in the dairy cattle industry had a higher K score increase post training than those involved in other industries, particularly sheep and goats. Dairy farming is a labor-intensive, capitally-intensive industry, offering considerable benefits to those who do it well, and milk production closely related to cow welfare, whereas sheep farming is extensive and offers less financial benefit to welfare improvement. Hence, it is possible that those drawn to these industries have different levels of motivation for knowledge improvement, with dairy farmers likely to benefit most.

The study highlighted the strong differences in religion between the different countries, with a contrast between two countries with high religiosity, Malaysia and Thailand, and two with low levels, China and Vietnam. In addition, Malaysia and Thailand had different dominant faiths, Islamism and Buddhism, respectively. Despite these strong contrasts, religion did not influence knowledge or the acquisition of knowledge in the training sessions. It could be hypothesized that the religious beliefs of Malaysian participants may have hindered them from, for example, improving their knowledge of the pig welfare standards, since 60% of respondents were Muslims, similar to 61% of Malaysians overall [33]. However, there was no evidence of this. Islamic doctrine advocates prevention of unnecessary suffering of any animal before and during slaughter [34]. Malaysia is among the most religious countries in the world [35], whereas China and Vietnam are amongst the least, and this was reflected in the survey respondents’ beliefs—67% of respondents claimed not to follow any religion, the same proportion as has been recorded nationally [36]. Agriculture is very important to both of the less religious countries, China and Vietnam (44% of the working population in Vietnam are employed in agriculture according to 2015 statistics [37]).

Thai respondents showed the lowest level of knowledge and were more likely to indicate that they thought they had little knowledge, but this is likely to reflect the fact that the stakeholders were younger and more likely to have less than one year of experience than in other countries. They showed the most significant improvement, with a mean difference of +33.9% after the workshops. Thailand’s agriculture sector is also very important, employing over 70% of the working population [38] and it has a particularly strong dairy industry, which is used for social support [39,40]. As the respondents associated with the dairy industry improved their knowledge more than those in other industries, as did those from Thailand, compared with the other countries, further training sessions to livestock stakeholders regarding the OIE animal welfare standards would probably be of substantial benefit to Thai stakeholders involved in the slaughter and transport of dairy cattle. The Thai government may be supportive; in 2016, it introduced ”Thailand 4.0”, an economic model which aims to achieve a 7-fold increase in the average annual income of farmers from 56,450 baht (5,470 USD) to 390,000 baht (15,000 USD) by 2037 [41].

## 7. Attitude Effects on Knowledge Score

People that were uncertain about improving animal welfare generally, the influence of the law and their company, and the limitations of their knowledge actually had less knowledge. This suggests a common approach in some respondents of not considering their beliefs and not attempting to acquire knowledge. This could reflect a lack of incentive or capacity to make change [26]. Conversely, uncertainty about the importance of vehicle design, a much more tangible topic, was associated with higher K scores. This may be because the respondents were not livestock transporters and a recognition of this uncertainty appears to have been more common amongst more knowledgeable respondents. Understanding the impact of vehicle design would be limited in those in other professions. Similarly, uncertainty about the importance of encouragement by their supervisor appears to have been more highly associated with a high level of knowledge. Thus the personal influence of the supervisor appears to be differently perceived to that of the company’s influence, and appears potentially more influential on knowledge, whereas the company’s influence is generally antagonistic to knowledge. Those who were uncertain or agreed that the supervisor was influential had more knowledge. For knowledge improvement, there was evidence that those with uncertainty about the importance of peers and knowledge on their ability to make improvements, or uncertainty about whether welfare of transported animals was satisfactory had greater improvement. This is to be expected because, at least for knowledge and law importance, the scores were lower in the first assessment; therefore, they were likely to rise more in the second. Similarly, those uncertain about the importance of vehicle design had greater scores in the first assessment and they increased less than those with firm views in the second.

Overall, there was little impact of knowledge on attitudes to animal welfare, in agreement with a recent study on the effects of knowledge of meat production systems on attitudes of people in Brisbane, Australia, towards chicken welfare and consumption, which concluded that increasing knowledge of the industry does not necessarily increase empathy towards animal welfare [21].

## 8. Demographic Effects on Knowledge Score and Its Improvement

Gender was not an apparent barrier to knowledge. Wambui et al. 2018 [42] reported that female stockpeople, aged over 50, and with livestock experience greater than 10 years had a significantly higher level of animal welfare knowledge, which is reflected in their attitudes and livestock practices.

The workshops highlighted the fact that significant improvement in animal welfare understanding can be achieved, which may lead to improved behavior in interactions with animals. Coleman and Hemsworth (2014) [43] found that training had the capacity to improve stockpersons’ beliefs and behaviors towards enhancing animal welfare. Our study also suggested that ethical and cultural backgrounds, rather than necessarily people’s religion, must be considered in designing training programs for the region. They should highlight the commercial advantages for each country to adopt animal welfare practices as well as the forecast economic future of a progressive advancement that is anticipated for the region. The major importance of country in the study suggests benefit from the integration of local communities to develop specific tailored training to deliver successful programs to improve the understanding of the OIE animal welfare standards in relation to livestock transport and slaughter practices. The tailored training programs should take into consideration the cultural and socioeconomic measures and encourage local relevant bodies to take responsibility for monitoring the agreed program.

Formal training was an important predictor of knowledge score improvement, indicating that this provided an improved ability to learn. It would be, therefore, desirable to the students in current university agriculture, veterinary studies and other animal related fields in these countries to receive a compulsory animal welfare and ethics syllabus to ensure that they have a good understanding of contemporary welfare issues.

The feedback received from participants in the workshops identified that improved knowledge of the OIE animal welfare standards during transport and slaughter by training is achievable, effective, well received by locals in the four East and Southeast Asian countries and forms a future opportunity for the OIE in spreading animal welfare standards in the region. Although the animal welfare concept is relatively new to the region [44], there are encouraging steps which have been taken by the four countries. Thailand has passed a Prevention of Cruelty and Animal Welfare Provision Act 2014 [45] and Malaysia has developed an Animal Welfare Bill that has recently been enacted [46]. Currently, there is no animal welfare legislation in Vietnam, or national animal welfare legislation in China [47,48]; however, some animal protection control was introduced in China in September 2009 [49].

Improving knowledge of the OIE recommendations during slaughter and transport has become a pre-requisite for any future improvement of animal welfare practices in these four east and southeast Asian countries, which may facilitate a change of attitude towards animal welfare [27,28]. Our research shows that improved knowledge will help people to define their attitudes to welfare issues. Other research has suggested behavior improvements as well; a study by Hemsworth (2003) [50] suggested that cognitive-behavioral intervention training programs designed to specifically target livestock stakeholders would have a direct effect on animals’ level of fear, welfare and productivity. 

## 9. Conclusions

Animal welfare improvement in the four Southeast Asian countries should focus on:

(a) Improving the OIE animal welfare standard and husbandry knowledge of livestock industry stakeholders.
(b) Local research and training programs based on moral and ethical concepts about animal welfare during slaughter and transport. The education programs should be aimed at all age groups.
(c) Adopting local public animal welfare awareness campaigns aimed at students in education, multimedia platforms and social organizations, which will bring about improvements in knowledge about animal welfare.

## Figures and Tables

**Table 1:** Number of participants in the initial ‘train the trainer’ and livestock stakeholders’ workshops.

	Malaysia	China	Vietnam	Thailand	Total Participants
Trainers	30	46	20	22	118
Stakeholders	94	338	196	268	896
Total	124	384	216	290	1014

**Table 2:** Number of participants in the livestock stakeholders’ workshops in each country.

Country	Locations of the Workshops
Malaysia	Zon Selatan, Tengah, Utara, Sabah, Sarawak, Pantai Timiur and Kuala Lumpur
China	Guandong, Hain, Hubei, Hun, Shandong, Zhejiang and Jiangxi
Vietnam	Hanoi, Halphong, Vinh, Dang, Vungtau, Binhduong and Cantho
Thailand	Khon Ratchasima, Udon Thani, Champon, Khon Kaen, Sakon Khon, Petchaburi and Bangkok

**Table 3:** Demographic characteristics of respondents (*n* = 1014) in Malaysia, China, Vietnam and Thailand.

Demographic	Respondents, *n* (% of Total Responses within Country)			
	Malaysia	China	Vietnam	Thailand
Total	124 (12)	384 (38)	216 (21)	290 (29)
**Gender**				
Male	90 (73)	294 (77)	157 (73)	142 (49)
Female	30 (24)	87 (23)	53 (25)	140 (48)
No answer	4 (3)	3 (1)	6 (2)	8 (3)
**Residential zone**				
Rural	60 (48)	121 (32)	87 (40)	148 (51)
Urban/metropolitan	59 (48)	258 (67)	123 (57)	135 (47)
No answer	5 (4)	6 (1)	6 (3)	7 (2)
**Age**				
Under 25	7 (6)	50 (13)	13 (6)	81 (28)
26–35	47 (38)	157 (41)	99 (46)	52 (18)
36–45	26 (21)	117 (30)	64 (30)	72 (25)
46–55	29 (23)	50 (13)	29 (13)	41 (14)
56–65	11 (9)	2 (0.5)	3 (1)	29 (10)
Over 65	1 (1)	5 (1.3)	1 (1)	7 (2)
No answer	3 (2)	3 (0.8)	7 (3)	8 (3)
**Religion**				
Buddhist	15 (12)	47 (12)	69 (32)	280 (97)
Atheist/don’t follow religion	0 (0)	259 (67)	134 (62)	0 (0)
Muslim	74 (60)	1 (0)	0 (0)	2 (1)
Christian	23 (18)	10 (3)	3 (1)	2 (1)
Other	12 (10)	71 (18)	10 (5)	6 (2)
**Religiosity**				
Not religious at all	2 (2)	193 (51)	106 (49)	31 (11)
Not very religious	5 (4)	63 (16)	64 (30)	66 (23)
Moderately religious	70 (57)	109 (28)	21 (10)	176 (61)
Very religious	40 (32)	16 (4)	5 (2)	12 (4)
No answer	7 (5)	3 (1)	20 (9)	5 (1)
**Job role**				
Work with animals	33 (27)	150 (39)	45(21)	76 (26)
Team Leader	24 (27)	61 (16)	11 (5)	30 (10)
Business owner	2 (1)	11 (3)	3 (1)	20 (7)
Business manager	11 (9)	41 (11)	5 (2)	9 (3)
Farmer	7 (6)	6 (2)	14 (7)	130 (45)
Practicing veterinarian	11 (9)	94 (25)	28 (13)	13 (5)
Veterinary advisor	19 (15)	17 (4)	101 (47)	7 (2)
No answer	7 (6)	4 (1)	9 (4)	5 (2)
**Level of industry understanding**				
Expert	2 (2)	56 (15)	1 (1)	2 (0.7)
Good knowledge	24 (20)	121 (32)	56 (26)	51 (18)
Some knowledge	55 (44)	151 (39)	106 (49)	136 (47)
Little knowledge	36 (29)	30 (8)	32 (15)	80 (28)
No knowledge	3 (2)	21 (5)	6 (2)	14 (5)
No answer	4 (3)	5 (1)	15 (7)	7 (2)
**Knowledge acquisition**				
Formal qualifications	43 (35)	206 (54)	104 (48)	111 (38)
Farm Employment	35 (28)	63 (16)	1 (1)	43 (15)
Personal interest	13 (10)	37 (10)	7 (3)	35 (12)
Friends	5 (4)	9 (2)	2 (1)	54 (19)
All of the above	22 (18)	60 (16)	83 (38)	35 (12)
No answer	6 (5)	9 (2)	19 (9)	12 (4)
**Type of livestock involvement**				
Beef/buffalo production	27 (22)	22 (6)	15 (7)	66 (7)
Dairy industry	8 (6)	12 (3)	2 (1)	30 (10)
Abattoirs/meatworks	26 (21)	151 (39)	87 (40)	27 (9)
Sheep/goat meat production	13 (11)	10 (3)	0 (0)	29 (10)
Wool/hair production	0 (0)	4 (1)	0 (0)	2 (1)
Poultry industry	25 (20)	59 (15)	15 (7)	62 (22)
Meat processing	2 (2)	14 (4)	6 (3)	10 (3)
Pig production	9 (7)	108 (28)	24 (11)	52 (18)
No answer	14 (11)	4 (1)	67 (31)	12 (4)
**Years of industry involvement**				
Up to 1 year	9 (7)	62 (16)	6 (3)	74 (26)
2–3 years	20 (16)	59 (15)	28 (13)	51 (18)
3–5 years	20 (16)	76 (20)	29 (13)	44 (15)
5–9 years	28 (23)	71 (19)	57 (26)	28 (10)
10–15 years	11 (9)	59 (15)	41 (19)	40 (14)
Over 15 years	27 (22)	53 (14)	39 (18)	30 (10)
No answer	9 (7)	4 (1)	16 (8)	23 (8)

**Table 4:** Significant (*p* < 0.05) effects of attitudes to the welfare of animals during transport and slaughter on Knowledge Scores (SED = 0.221).

Attitude Question	Strongly Disagree	Disagree	Neither Agree nor Disagree	Agree	Strongly Agree	F-Value	*p*-Value
I intend to make improvements to welfare of animals in my care	4.62 ^ab^	2.89 ^ab^	2.54 ^b^	3.17 ^a^	3.38 ^a^	2.61	0.03
The laws on animal slaughter and transport influence my assessment of their AW at this time	4.02 ^a^	2.99 ^ab^	2.63 ^b^	3.33 ^a^	3.62 ^a^	4.30	0.002
My knowledge about animal slaughter and transport limits my ability to improve AW during transport	3.46 ^ab^	2.56 ^b^	2.84 ^b^	3.14 ^b^	3.91 ^a^	2.34	0.04
Vehicle design makes improvement to AW during transport hard	2.34 ^c^	3.34 ^bc^	4.07 ^a^	3.74 ^ab^	3.10 ^bc^	3.68	0.006
Changes prescribed by my company encourage me to change practices	5.52 ^a^	3.20 ^b^	2.27 ^c^	2.56 ^bc^	3.03 ^b^	4.40	0.002
Changes prescribed by my supervisor encourage me to change practices	2.43 ^b^	3.56 ^ab^	3.90 ^a^	3.95 ^a^	2.75 ^b^	3.87	0.004

AW = animal welfare. Means with different superscripts (a, b or c) within rows are significantly different (*p* < 0.05) by Tukey’s test.

**Table 5:** Significant (*p* < 0.05) effects of attitudes to the welfare of animals during transport and slaughter on improvement in Knowledge Scores after training (SED = 0.300).

Attitude Question	Strongly disagree	Disagree	Neither Agree nor Disagree	Agree	Strongly Agree	F-value	*p*-Value
Welfare of transported animals is satisfactory	5.90 ^ab^	5.07 ^b^	6.04 ^a^	5.30 ^b^	5.20 ^ab^	2.38	0.05
Importance to my peers of factors influencing welfare of animals	5.52 ^abc^	4.88 ^bc^	6.53 ^a^	5.78 ^b^	4.79 ^c^	4.81	0.001
Company approval towards improving the welfare of animals	5.94 ^ab^	5.74 ^a^	4.50 ^b^	5.28 ^a^	6.03 ^a^	2.92	0.02
Vehicles design influences ability for improvement	7.03 ^a^	5.75 ^a^	4.34 ^b^	4.65 ^b^	5.73 ^a^	5.46	0.001
Encouraged to change if prescribed by government	3.95 ^b^	5.62 ^b^	6.74 ^a^	5.66 ^b^	5.54 ^b^	3.85	0.004
Encouraged to change if prescribed by law	7.31 ^a^	4.59 ^bc^	4.90 ^c^	4.87 ^c^	5.83 ^ab^	2.83	0.02
Monetary gain influences my personal assessment of welfare	6.61 ^a^	5.27 ^bc^	4.79 ^c^	5.48 ^ab^	5.36 ^abc^	2.95	0.02
Importance of welfare to peers influences ability to make improvement during slaughter	1.04 ^c^	7.39 ^a^	6.49 ^ab^	6.69 ^ab^	5.89 ^b^	3.84	0.004
My personal beliefs influence my ability to make improvement during transport	5.41 ^ab^	6.07 ^a^	5.87 ^a^	5.86 ^a^	4.29 ^b^	2.61	0.03

Means with different superscripts (a, b or c) within rows are significantly different (*p* < 0.05) by Tukey’s test.

## Supplementary Materials

The following are available online at https://www.mdpi.com/2076-2615/9/3/99/s1, Questionnaire Section 1: Descriptive statistics are provided for each husbandry knowledge question in the stakeholders workshops, Questionnaire Section 2: Attitudes towards livestock management, with special emphasis on transport and slaughter, Questionnaire Section 3: Demographic Background.
