# Supplementary material for: Knowledge of Stakeholders in the Livestock Industries of East and Southeast Asia about Welfare during Transport and Slaughter and Its Relation to Their Attitudes to Improving Animal Welfare
Source: Animals (Basel). 2019 Mar 19;9(3):99. doi: 10.3390/ani9030099 (PMC6466319; doi:10.3390/ani9030099)
Supplement: Supplementary file 1 [file animals-09-00099-s001.pdf]

The questionnaire

Section 1

The purpose of this section is to find out about how effective the current roadshow presentations will increase the level of knowledge and understanding of OIE standards regarding the welfare of livestock in South East Asian countries. This section will focus on your Knowledge on animal production systems in particular during Slaughter and Transport.

Your responses will only be identifiable to you by coding. Please feel free to decline to answer any of the questions at any time or to withdraw from the survey. The answers will be coded by a unique number given to individual participants ending with "B" or "A" which stand for before and after the training takes place.

Descriptive statistics are provided for each husbandry knowledge question in the stakeholders workshops. Correct answers are indicated in bold. X_1 = mean husbandry knowledge scores before training in %, X_2 = mean husbandry knowledge scores after training in %, D = mean difference of husbandry knowledge scores after training, in %).

	Question	X_1%	X_2%	D%
Q1	When taking a laying hen out of a multiple bird cage:<br>• hold bird firmly by the neck and tail and remove from cage slowly with bird facing towards the door<br>• grasp bird by one leg and the comb and remove from cage slowly with bird facing backwards<br>• grasp bird by shanks of both legs with one hand and support breast with other hand. Flip onto back before withdrawing from cage with head towards the door<br>• **grasp shanks of both legs with one hand and support breast with other hand. Withdraw slowly from cage with bird facing backwards**<br>• grasp right wing and left leg and remove slowly from cage with bird facing backwards	27.9	65.2	+37.3
Q2	Is it normal practice in your country for livestock to be rendered unconscious (stunned) before being killed?<br>• yes<br>• no/don't know	26.5	35.0	+8.5
Q3	The organisation that sets international standards for livestock transport and slaughter is:<br>• Royal Society for Prevention of Cruelty to Animals (RSPCA)<br>• World Animal Protection<br>• **World Animal Health Organisation (OIE)**<br>• World Society for Protection of Animals (WSPA)<br>• Food and Agriculture Organisation of the United Nations (FAO)	32.4	77.7	+45.3
Q4	Pigs are prone to heat stress during transport; they naturally avoid heat stress by:<br>• panting<br>• dust bathing<br>• **wallowing**	20.9	44.9	+24.0

	• huddling<br>• burrowing			
Q5	Which of the following gases can cause problems in transport, especially in ships that are inadequately ventilated?<br>• **ammonia**<br>• carbon dioxide<br>• carbon monoxide<br>• hydrogen sulphide<br>• all of the above	17.0	61.3	+44.3
Q6	Which of the following is incorrect in relation to handling races for cattle?<br>• **curved races are more likely to make cattle baulk compared with straight ones**<br>• narrow shafts of light make cattle stop moving<br>• they should be wide enough to allow the animal to move freely but not turn around<br>• a solid side facilitates movement<br>• the floor should be non-slip	13.9	44.4	+30.5
Q7	What is the maximum angle of a loading ramp into a vehicle or ship?<br>• 10°<br>• **20°**<br>• 25°<br>• 30°<br>• 35°	15.2	59.2	+44.0
Q8	When selecting cattle for slaughter using teeth eruption for ageing cattle, which of the following statements is correct?<br>• six permanent incisors erupt at 30–42 months<br>• six permanent incisors erupt at 12–24 months<br>• six permanent incisors erupt at 24–36 months<br>• **six permanent incisors erupt at 40 months**<br>• none of the above	7.6	49.1	+41.5
Q9	By using a body condition score to select animals for slaughter we are describing which of the following:<br>• the immediate health status of livestock<br>• the amount of fat tissue in livestock<br>• **the amount of fat and muscle tissue in livestock**<br>• the level of contents of the digestive tract in livestock<br>• the amount of muscle tissue	23.9	54.0	+30.1
Q10	To move pigs effectively, the best method to use is:<br>• a collar around the neck with a leash	34.7	88.5	+53.8

	• a halter around the head ……….<br>• **a pig board to block the pigs and a paddle to guide them in the desired direction**<br>• a whip<br>• a cattle prodder			
Q11	When selecting sheep for slaughter by looking at their teeth, what is the approximate age of a sheep with two permanent incisor teeth?<br>• 6 months<br>• **12 months to 18 months**<br>• 2 years<br>• 3 years<br>• 4 years	29.0	54.0	+25.0
Q12	Which one of the following is not a result of spending time off food and water during transport?<br>• hunger<br>• reduction in rumen microbial activity<br>• potential increase in enteropathogenic bacteria<br>• mobilising energy from fat stores<br>• **weight gain of 10–15%**	36.4	74.1	+37.7
Q13	Which one of the following is not a means of determining how many animals to put on a truck?<br>• weight of the animal<br>• fleece length in sheep<br>• the animals' pregnancy status<br>• **length of the journey**<br>• whether the animal have horns or not	14.6	49.6	+35.0
Q14	For cattle and sheep, heat stress occurs above what temperature?<br>• 15–19 ^{o}C<br>• 20–25 ^{o}C<br>• **26–30 ^{o}C**<br>• 31–35 ^{o}C<br>• 36 ^{o}C	14.7	52.3	+37.6
Q15	Which of the following is not a result of stress in animals before slaughter?<br>• bruising<br>• bone breakage<br>• **loss of carcass weight**<br>• pale soft exudative (PSE) meat<br>• dark firm dry (DFD) meat	19.3	50.0	+30.7

Q16	Cattle droving: when taking cattle to market during good weather conditions, what it the maximum distance that well fed, fit cattle can be driven in one day?<br>• 5 km<br>• 10 km<br>• 15 km<br>• **30 km**<br>• 50 km	10.9	46.4	+35.5
Q17	Which of the following is not a method used for stunning animals prior to slaughter?<br>• **hitting against a solid wall for piglets**<br>• captive bolt for cattle<br>• electrical tongs for sheep and pigs<br>• electrified water bath for chickens<br>• gas stunning by carbon dioxide for chickens and pigs	39.5	65.6	+26.1
Q18	Approximately what output in chickens per hour can a typical modern chicken slaughter and processing facility produce?<br>• 100 birds<br>• 500 birds<br>• **4000 birds**<br>• 8000 birds<br>• 15000 birds	30.4	52.2	+21.8

Section 2. Attitudes towards livestock management, with special emphasis on transport and slaughter

Part A

'The welfare of animals' means how an *animal* is coping with the conditions in which it lives. An *animal* is in a good state of *welfare* if (as indicated by scientific evidence) it is healthy, comfortable, well nourished, safe, able to express innate behaviour and is not suffering from unpleasant states such as pain, fear and distress (World Organisation for Animal Health -**OIE**).

'Community' refers to a social group of any size whose members reside in a specific locality, share government, and often have a common cultural and historical heritage.

	Strongly Disagree	Disagree	Neither Disagree or Agree	Agree	Strongly Agree
**1.** The welfare of the animals during slaughter is important to me					
**2.** The welfare of the animals during transport is important to me					
**3.** The welfare of the animals while being slaughtered is satisfactory in my workplace					
**4.** The welfare of the animals while being transported is satisfactory in my workplace					
**5.** Most people who are important to me would approve of me making improvements to the welfare of the animals in my care					
**6.** I intend to make improvements to the welfare of the animals in my care					
**7.** I am confident that I can make improvements to the welfare of animals					
**8.** In the past I have tried to make improvements to the welfare of the animals in my care					
**9.** The following factors influence **my personal** assessment of the welfare of the animals during slaughter and transport;					
9a. My religious beliefs					
9b. My personal beliefs (not religious)					
9c. The extent to which there are more pressing concerns than the welfare of animals in my community					
9d. My monetary gain					
9e. Monetary gain to my community					
9f. How important the welfare of animals is to the company I work for					
9g. How important it is to my peers					
9h. My knowledge about animal slaughter and animal transportation practices					
9i. The laws relevant to animal slaughter and animals transportation practices					
**10.** The following factors influence **my communities'** assessment of animal welfare standards during slaughter and transport					
10a. Religious beliefs					
10b. Personal beliefs (not religious)					
10c. The extent to which there are more pressing issues than the welfare of animals in their community					
10d. Monetary gain for themselves					
10e. Monetary gain to the company they work for					
10f. How important the welfare of animals is to the company they work for					

10g. Their knowledge about animal slaughter and transportation practices					
10h. The government laws and monitoring relevant to animal slaughter and transportation practices					
**11.** The main factors that influence my ability to make improvements to animal welfare during **slaughter** include the following:					
11a. My religious beliefs					
11b. My personal beliefs (not religious)					
11c. The extent to which there are more pressing concerns than the welfare of animals in my community					
11d. Monetary gain to myself					
11e. Monetary gain to my community					
11f. How important the welfare of animals is to the company I work for					
11g. How important the welfare of animals is to my peers					
11h. Company approval towards improving the welfare of animals					
11i. My knowledge about animal slaughter and animal transportation practices					
11j. My work space					
11k. The availability of tools and resources					
11l. The government laws and monitoring relevant to animal slaughter and transportation practices					
**12.** The main factors that influence my ability to make improvements to animal welfare during **transport** include the following:					
12a. My religious beliefs					
12b. My personal beliefs (not religious)					
12c. The extent to which there are more pressing concerns than the welfare of animals in my community					
12d. Monetary gain to myself					
12e. Monetary gain to my community					
12f. How important the welfare of the animals is to the company I work for					
12g. How important the welfare of the animals is to my peers					
12h. Company approval towards improving the welfare of animals					
12i. My knowledge about animal slaughter and animal transportation practices					
12j. My work space					
12k. The vehicles I use are designed in way that makes it very hard to make improvements					
12l. The availability of tools and resources					
12m. The government laws and monitoring relevant to animal slaughter and animal transportation practices					
**13.** I am more encouraged to change my practices if;					
13a. Changes are prescribed by local government					
13b. Changes are prescribed by a local organization					
13c. Changes are prescribed by local law enforcement (police)					
13d. Changes are prescribed by a western international organization					
13e. Changes are prescribed by law					
13f. Changes are prescribed by my company					
13g. Changes are prescribed by my supervisor					
13h. Changes are prescribed by my community elder or community leader					
13i. I see moral value in changing practices					
13j. I see personal monetary gain from changing practices					
13k. I see others making the changes					

Section 3: Demographic Background

1. Please indicate your gender;

a. Male………………………………………………………………………. □

b. Female…………………………………………………………………. □

c. Transgender………………………………………………………… □

2. In what type of area have you lived for most of your life?

a. Rural……………………………………………………………………. □

b. Urban…………………………………………………………………… □

c. Metropolitan……………………………………………………….. □

3. Are you happy to tell me your marital status?

a. Single, no children……………………………………………… □

b. Single, children…………………………………………………. □

c. Married/De Facto, no children……………………………… □

d. Married/De Facto, children……………………………….…. □

e. Widowed…………………………………………………………. □

4. Please indicate your age range;

a. Under 18……………………………………………………………….. □

b. 18–25……………………………………………………………..…….. □

c. 26–35…………………………………………………………………… □

d. 36–45……………………………………………………………..…….. □

e. 46–55…………………………………………………………………… □

f. 56–65………………………………………………………………………… □

g. Over 65………………………………………………………….………… □

5. Which religion do you follow?

a. Bahai' Faith…………………………………………………………. □

b. Buddhism……………………………………………………………… □

c. Caodaism……………………………………………………………. □

d. Chinese folk religion…………………………………………….... □

e. Chondogyo…………………………………………………………….. □

f. Christianity……………………………………………………………… □

g. Confuciansim………………………………………………………… □

h. Hinduism………………………………………………………………… □

i. Islam…………………………………………………………………………… □

j. Jainism………………………………………………………………………. □

k. Judaism………………………………………………………………….□

l. Shinto………………………………………………………………………. □

m. Sikhism………………………………………………………………….. □

n. Taoism………………………………………………………………… □

o. I don't' follow a religion…………………………………………………. □

p. Other (please specify) ______________________________ □

6. **To what extent do you consider yourself religious?**

a. Not religious at all……………………………………………………….. □

b. Not very religious………………………………………………………….. □

c. Moderately religious…………………………………………………….. □

d. Very religious……………………………………………………….…..□

7. **What region of your country do you belong to?**

Malaysia –

a. West……………………………………………………………………… □

b. East (Sabah and Sarawak)……………………………………….… □

China –

a. South……………………………………………………………………… □

b. Central……………………………………………………………………. □

c. North……………………………………………………………………… □

d. East……………………………………………………………………. □

e. Northeast………………………………………………………………… □

f. Northwest…………………………………………………………...……… □

g. Southwest……………………………………………………...……… □

h. Hong Kong Macao area……………………………………...………□

Thailand –

a. North (Upper)……………………………………………………………. □

b. North (Lower)…………………………………………………………….. □

c. Northeast (Eastern)…………………………………………………… □

d. Northeast (Upper)…………………………………………….……….. □

e. Northeast (Lower)……………………………………………………. □

f. Central……………………………………………………………………… □

g. East……………………………………………………………………. □

h. West……………………………………………………………………… □

i. South (Upper) …………………………………………………………..□

j. South (Lower)……………………………………………………………….. □

Vietnam –

- a. North…………………………………………………….………………….. □
- b. South…………………………………………………….………………….. □
- c. Central (including Highland)……………………………….…………….. □

8. Please indicate which job role best describes your involvement in the animal transport and slaughter industries

- a. Work directly with the animals……………………………………………. □
- b. Team Leader: Supervise people who work directly with the animals… □
- c. Business owner…………………………………………………………….. □
- d. Business Manager………………………………………………………. □
- e. Farmer……………………………………………………………..…… □
- f. Veterinarian who treats animals hands on…………………………………… □
- g. Veterinarian working for the Government as an advisor……………..…. □

9. Please indicate the type of livestock industry you are involved with

- a. Beef cattle and buffalo production……………………………………... □
- b. Dairy industry (cattle/sheep/goat/buffalo). ……………………………….. □
- c. Abattoirs/meatworks……………………………………….…….……. □
- d. Sheep and goat meat production……………………………..…….…. □
- e. Sheep wool and goat hair Production…………………….…………… □
- f. Poultry Industry (Broiler / Egg / Feed / Breeding / Hatchery) …… □
- g. Meat processing…………………………………………………….……… □
- h. Pig Production…………………………………………………..….…. □

10. Please indicate your level of understanding of livestock production systems

- a. Expert…….……………………………………………………………..… □
- b. Good knowledge……..………………………………………………. □
- c. Some knowledge……..……………………………………………… □
- d. Little knowledge…………………………………………..………..…. □
- e. No knowledge ……………………………………………………….….. □

12 How did you gain your knowledge?

- a. Formal qualifications—relevant degree, training course…………… □
- b. Farm employment—hands on experience…………………………… □
- c. Personal interest—internet, journals, newspaper articles, and television programmes…□
- d. Friends and acquaintances……………………………………………. □
- e. All of the above……………………………………………………..…. □

2. Please indicate how long you have been working in the industry.

- a. Less than 1 year………………………………………………….….. □
- b. 2–3 Years………………………………………………………………… □
- c. 3–5 Years……………………………………………………….…... □
- d. 5–9 Years…………………………………………………….…… □

e. 10–15 Years……………………………………………………………..… □

f. More than 15 Years………………………………………..…………..… □

Please feel free to contact Professor Clive Phillips, c.phillips@uq.edu.au, **Centre for Animal Welfare and Ethics, School of Veterinary Science, University of Queensland, Gatton 4343, QLD, Australia, Tel. +61 7 5460 1158; mobile 0406340133,** if you have any questions or wish to comment on this survey.

Thank you for taking the time to complete this questionnaire, your opinions are greatly valued.
